# Autonomous Identification and Positioning of Trucks during Collaborative Forage Harvesting

**DOI:** 10.3390/s21041166

**Published:** 2021-02-07

**Authors:** Wei Zhang, Liang Gong, Suyue Chen, Wenjie Wang, Zhonghua Miao, Chengliang Liu

**Affiliations:** 1 School of Mechanical Engineering, Shanghai Jiao Tong University, Shanghai 200240, China; zhang_wei@sjtu.edu.cn (W.Z.); 020910287@sjtu.edu.cn (W.W.); chlliu@sjtu.edu.cn (C.L.); 2 School of Mechatronic Engineering and Automation, Shanghai University, Shanghai 200444, China; llsuchen@shu.edu.cn (S.C.); zhhmiao@shu.edu.cn (Z.M.)

**Keywords:** agricultural automation, forage harvester, collaborative unloading operation, identification and positioning, visual odometry, random sample consensus

## Abstract

In the process of collaborative operation, the unloading automation of the forage harvester is of great significance to improve harvesting efficiency and reduce labor intensity. However, non-standard transport trucks and unstructured field environments make it extremely difficult to identify and properly position loading containers. In this paper, a global model with three coordinate systems is established to describe a collaborative harvesting system. Then, a method based on depth perception is proposed to dynamically identify and position the truck container, including data preprocessing, point cloud pose transformation based on the singular value decomposition (SVD) algorithm, segmentation and projection of the upper edge, edge lines extraction and corner points positioning based on the Random Sample Consensus (RANSAC) algorithm, and fusion and visualization of results on the depth image. Finally, the effectiveness of the proposed method has been verified by field experiments with different trucks. The results demonstrated that the identification accuracy of the container region is about 90%, and the absolute error of center point positioning is less than 100 mm. The proposed method is robust to containers with different appearances and provided a methodological reference for dynamic identification and positioning of containers in forage harvesting.

## 1. Introduction

Under intensive rearing systems, the diets for dairy and beef cattle are generally offered as total mixed rations, rich in concentrated feedstuffs and roughage, to meet the daily nutrient requirements of the animal [1]. Roughage is not only an important source of energy for these ruminants, but also provides daily essential crude fiber. However, a sufficient amount of coarse fibrous ingredients in the diet is necessary to maintain normal ruminant function [2]. A lack of fiber can lead to stereotypes, nutritional disorders, and even ruminal acidosis [3,4]. Good quality silage, which is a major source of roughage, is produced from the green forage of corn, wheat, grass or other crops [5]. Compared to these green crops, silage is easier to store and transport, and its nutritional quality is more stable [6]. These advantages, together with low production costs, have greatly increased the demand for silage in modern intensive farms [7].

Forage harvesters are some of agriculture’s most versatile machines, capable of harvesting a variety of forage crops under different agronomic conditions [8]. Unlike the combine harvester that can temporarily store grains in the internal grain reservoir during harvesting operations, the forage harvester itself does not have a material holding tank for storing the harvested green forage. A common practice is that a pair of harvester and truck performs on-the-go unloading: the truck with a loading container needs to travel next to the harvester at a similar nonzero speed, continuously loading the picked-up and processed crop stream [9]. This kind of collaborative unloading process is cost-effective, but the implementation is more challenging, because the two moving vehicles need to be coordinated. In the course of this, the drivers of the forage harvester and the truck have to continuously monitor the unloading position of the forage stream, simultaneously adjusting the vehicle speeds and distances from each other so that the green forage does not fall on the ground [10,11]. At the same time, there are many other operations that require coordination by the drivers of agricultural machinery. For example, the driver must maintain the direction of the forage harvester, observe the status of the crop, monitor machine performance, confirm the fill level of the container, and pivot the discharge elbow manually, etc. [12]. Especially at the turning point, there is not enough space for the forage harvester and the truck to drive in parallel. The truck has to move behind the forage harvester. Thus, the change of the driving status will often cause mistakes and reduce work efficiency. Therefore, this continuous on-the-go unloading will distract the forage harvester’s driver, causing stress and fatigue [13]. Moreover, as working hour increase, it also could decrease harvest efficiency and affect operational safety. Due to the problems mentioned above, it is significant to investigate the automatic unloading of the forage harvester. The first task of this research is to solve the problem of identification and positioning of the moving truck container during collaborative operation. Only after the container is accurately identified and positioned, the forage harvester unloading system can control the unloading mechanism to make the silage fall in the ideal position, which also paves a way for autonomous driving for the machineries in the near future.

At present, the studies of automatic unloading of agricultural harvesting machinery are still in the initial stage. Meanwhile, there are very few references on the identification and positioning of the moving container during the automatic unloading of the forage harvester, and most of them are patents. Polklas [11] invented a sensor device for the automatic filling of containers, which consists of multiple optical and acoustic range finders. Since the container edges are much higher than its inner and outer sides, the height signal measured by the device will change significantly. In this way, the position of the container walls, as well as the filling level of forage in different positions in the container, can be identified. However, the measuring area of the range finder is limited. In order to measure complete information, the discharge elbow has to be rotated horizontally above the container until it covers the entire area. Alexia et al. [14] proposed a crop discharge spout control system based on two cameras. In order to prevent the forage stream from obstructing the field of view, two cameras were mounted on the end of the spout. They used image processing techniques to identify the hauling vehicle, such as low pass filtering, edge detection, thresholding, etc. In order to solve the problem of forage material loss and uneven unloading during manual unloading, Happich et al. [10] developed a prototype of an assistance system for overloading agricultural goods. The system used the Global Positioning System (GPS) to locate the forage harvester and the transport unit and allowed a closed-loop loading position control of the chute and deflector to unload the forage stream to a specific target point.

In the field of automatic unloading of grain combine harvesters, some technologies, including ultrasound, virtual reality and machine vision, have been initially applied to the identification and positioning of the loading container. Gaard [13] built an ultrasonic sensor package and developed an algorithm to model the container edges and the grain surface using distance data obtained by the sensor package. The algorithm can extract the features of the container and grain. However, the sensor package is sensitive to the installation cone angle, causing the error of the experimental results to be larger than expected. Kurita et al. [15] studied the automatic rice unloading problem and proposed a method to find the container and position the auger of the combine harvester at an appropriate point. This method used ARToolKit to detect a fiducial marker and calculate transform matrices between the marker’s and the camera’s coordinates. The size of the grain container is standard, and the positional relationship between the fiducial marker and the grain container is fixed. Therefore, the position of the container can be indirectly calculated by identifying the position and posture of the marker. Cho et al. [16] proposed a method for the autonomous positioning of the unloading auger and the grain container by a laser sensor and global navigation satellite system (GNSS). The method was sufficient for the successful positioning of the auger spout within the acceptable error range. Liu et al. [17] studied the identification of the grain loading situation in the grain container and proposed a method of parameter design of cellular neural network edge template based on ant lion algorithm to extract the edge of the grain container. After region segmentation, the grain loading status could be identified using color features. Finally, the effect of edge detection and grain status identification was verified under laboratory conditions.

In the process of green forage harvesting, the agricultural environment and crop objects are both unstructured [18,19,20]. A large amount of dust and forage particles will fall on the surface of the truck, obscuring the features of the container. Moreover, the size and shape of the container are not standard, and it is very common that the container is heightened or modified privately. Different from static unloading of the combine harvester, the forage harvester and the truck move independently in the three-dimensional (3D) space. Under this circumstance, the identification and positioning of the container need to be completed in a 3D coordinate system. These factors increase the difficulty of identifying and positioning the container of the forage transporter, which makes it difficult to achieve the expected results using only 2D image processing technology [13]. In order to realize the self-adaptive, non-marker identification and positioning of different types of containers in the unstructured agricultural environment, this paper establishes three coordinate systems to describe the system model of the forage harvester and the truck, and proposes a container-adaptive identification and positioning (CAIP) method based on depth vision. The method processes include data preprocessing, pose transformation of the point cloud based on the SVD algorithm, the upper edge segmentation of the container, the upper edge lines extraction and corner points positioning based on the RANSAC algorithm, and visualization of identification and positioning results on the depth image.

The remainder of this paper has been organized as follows. Section 2 establishes a global model with three coordinate systems to describe the collaborative harvesting system. The depth-vision-based CAIP method is then proposed, and the specific procedures of the proposed approach are also described in detail. In Section 3, field experiments on the identification and positioning of different containers are carried out, experimental results are analyzed and discussed, and the performance of the proposed approach is illustrated and verified. Finally, Section 4 gives conclusions and future work.

## 2. System Description and Proposed Method

### 2.1. System Description

**Figure 1:**
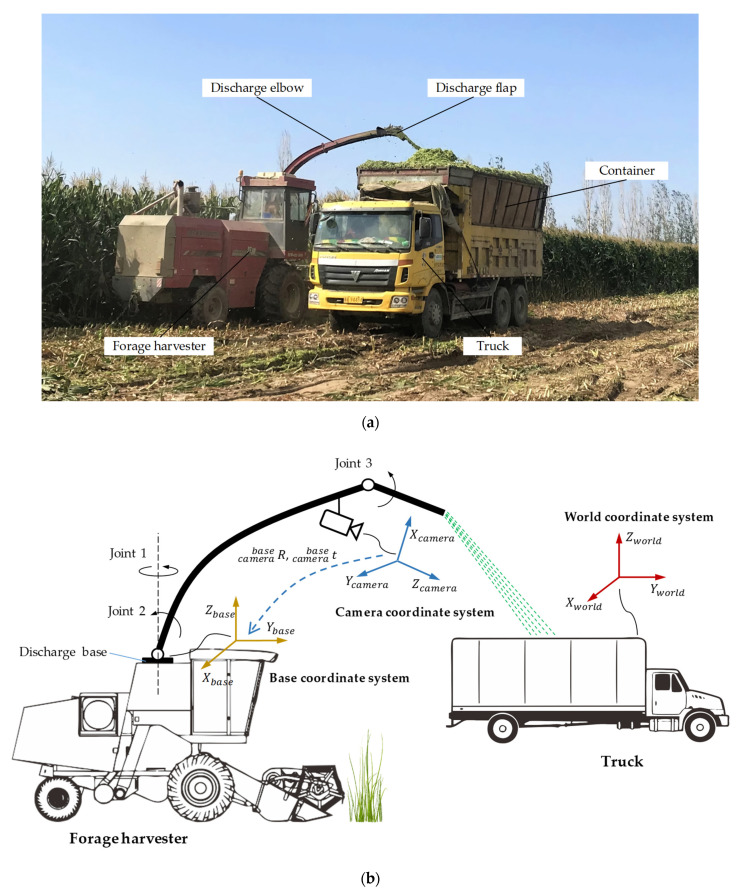
Collaborative unloading operation of the forage harvester and the truck. (**a**). Collaborative unloading operation site. (**b**). The simplified system model.

The traditional forage harvesting process is shown in Figure 1a. The forage harvester and the truck need to be closely coordinated. This is essentially a visual servocontrol problem, that is, the driver observes the position of the container, then determines the optimal unloading point of the forage stream, and finally, the mechanical device of the forage harvester can be controlled to accurately unload the forage to the target point until the container is evenly filled. The purpose of this paper is to accurately identify and position the moving container using depth vision and calculate the 3D coordinates of the unloading target point, which can provide feedback for the subsequent control of the unloading device of the forage harvester.

The simplified system model of the collaborative unloading operation of the forage harvester and the truck is shown in Figure 1b. The unloading device of the forage harvester consists of a discharge base, a discharge elbow, a discharge flap and three joints. Joint 1 is located on the discharge base, and it can control the discharge elbow to rotate around the rotation axis of the base. Joint 2 can adjust the elevation angle of the discharge elbow between 60° and 90°. Joint 3 can control the unloading angle of the discharge flap between 10° and 90°. In the harvesting process, due to the relatively large adjustable range of the discharge flap angle, the discharge elbow generally maintains the maximum elevation angle, and the unloading direction of the forage stream is usually controlled by the discharge flap. In order to obtain the best view, a color and depth (RGB-D) camera is fixed at a higher position of the discharge elbow.

**Figure 2:**
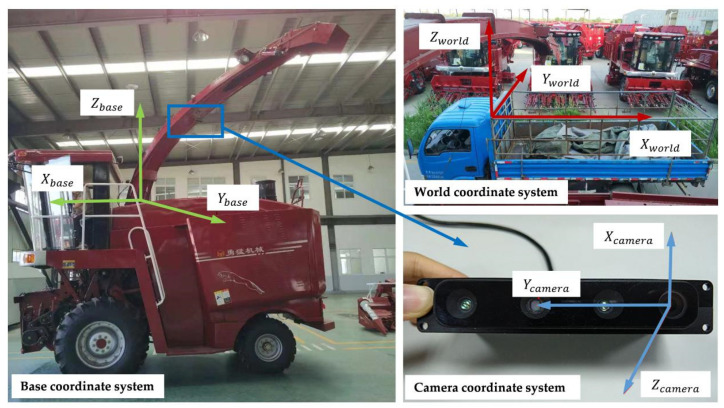
The corresponding positions of the coordinate systems in the real system.

To describe the system comprehensively, this paper has established three coordinate systems: world coordinate system, camera coordinate system and base coordinate system. The corresponding positions of these coordinate systems in the real system are shown in Figure 2.

The world coordinate system is established based on the upper edge of the container. The origin is located at the front left corner of the upper edge. The $X_{world}$ − $Y_{world}$ plane is parallel to the ground and coincides with the upper edge plane and the $Z_{world}$ axis is perpendicular to the ground.

The camera coordinate system is established based on the RGB-D camera, the RGB lens optical center on the right side of the camera is the origin of the camera coordinate system, the $X_{camera}$ − $Y_{camera}$ plane is parallel to the imaging plane, and the $Z_{camera}$ axis is the depth direction of the camera.

The base coordinate system is a dynamic coordinate system, which can follow the unloading device to rotate. Its coordinate origin is at the rotation center of the discharge base. The $Z_{base}$ axis is the rotation axis of the discharge base. The $X_{base}$ − $Y_{base}$ plane is parallel to the ground. The central axis of the discharge elbow is always located in the $Y_{base}$ − $Z_{base}$ plane. The $Y_{base}$ axis direction coincides with the projection direction of the unloading elbow on the $X_{base}$ − $Y_{base}$ plane. When the discharge base drives the discharge elbow to rotate around the $Z_{base}$ axis, the base coordinate system also rotates synchronously.

The camera coordinate system’s spatial position relative to the discharge elbow is fixed, so there is a constant transformation relationship between the camera coordinate system and the base coordinate system. The transformation relationship includes two parts: the rotation matrix Rcamerabase and the translation matrix tcamerabase. Since the truck and the forage harvester move independently, the transformation relationship between the base coordinate system and the world coordinate system is complicated and unknowable. However, the $X_{base}$ − $Y_{base}$ plane and the $X_{world}$ − $Y_{world}$ plane are parallel, and there is a constant and measurable height difference in the height direction.

### 2.2. Proposed Method

#### 2.2.1. Method Overview

There are two common features of the truck containers: (1) The container’s upper edge has obvious straight-line characteristics. (2) The upper edge of the container is located at the local highest position of the truck, that is, the z-axis values of the upper edge points are relatively large in the world coordinate system. These features will neither be changed due to the coverage or occlusion of the silage during unloading nor will it be affected by the size and type of different trucks. However, the point cloud of the truck acquired by the camera is based on the camera coordinate system. The camera coordinate system and the world coordinate system move independently, and there is no fixed transformation relationship. As described in Section 2.1, the $X_{base}$ − $Y_{base}$ plane and the $X_{world}$ − $Y_{world}$ plane are parallel, and both are parallel to the ground. In the base coordinate system, the feature that the z-axis values of the upper edge points are relatively large will not change. Therefore, a reasonable approach is to transform the point cloud of the truck to the base coordinate system using the transformation matrix from the camera coordinate system to the base coordinate system, and then extract and locate the upper edge of the container in the base coordinate system according to the above-mentioned features.

**Figure 3:**
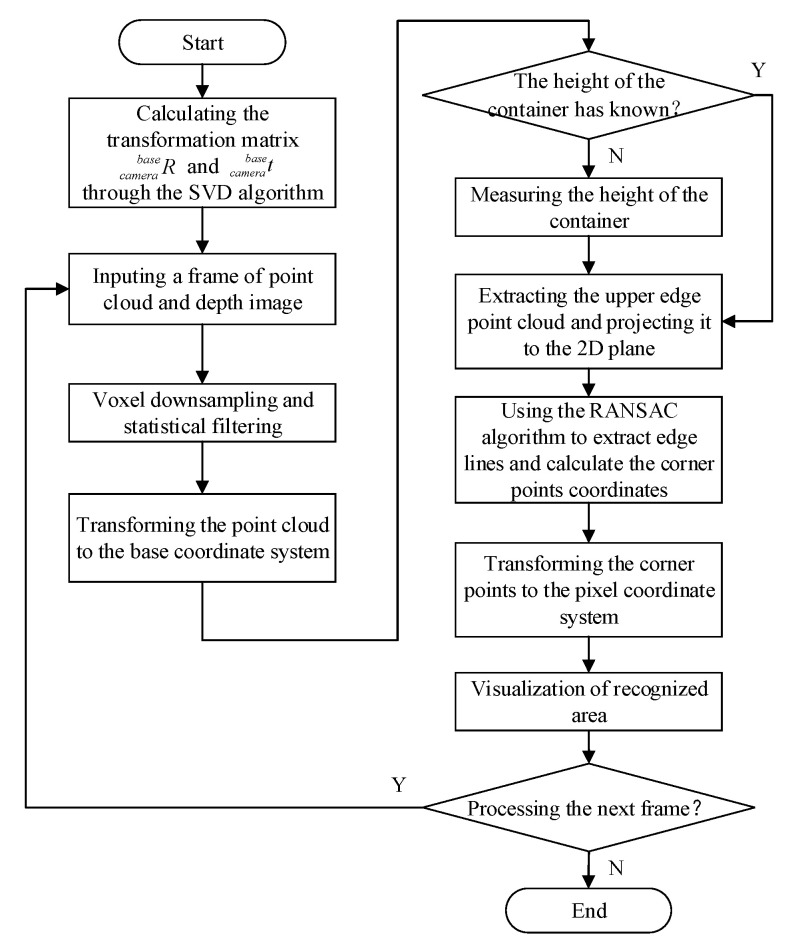
The flow chart of the depth-vision-based CAIP method.

Based on the above description, the depth-vision-based CAIP method is proposed to identify and position the truck container. First, the RGB-D camera is used to obtain point cloud images, RGB images and depth images of the truck, and then the point cloud data is preprocessed to compress the number of points and remove noisy points, including voxel downsampling and statistical filtering. Second, the SVD algorithm is used to solve the pose transformation matrix from the camera coordinate system to the base coordinate system, and the point cloud is converted to the base coordinate system. Subsequently, the container’s upper edge points are segmented through the threshold processing of the z-axis value and then projected into a 2D image. Finally, the upper edge lines and corner points are identified and positioned based on the RANSAC algorithm and then visualized on the depth image. The flow chart of the depth-vision-based CAIP algorithm is shown in Figure 3, and the implementation process will be described in detail following the steps of the method.

#### 2.2.2. Point Cloud Preprocessing

The amount of point cloud data captured by the RGB-D camera is generally very large. In this paper, the number of points per frame is more than 200,000. The point cloud data downsampling is necessary to reduce the density and improve the calculation efficiency but preserving as much original information as possible. The VoxelGrid is an effective 3D point cloud downsampling technique, which can reduce the number of points while keeping the contour of the point cloud unchanged [21,22]. The VoxelGrid algorithm defines a constant-size voxel grid to decompose the 3D space of the input point cloud into multiple 3D voxels. After the decomposition operation, each voxel is a small point set containing a different number of points. For each voxel, a centroid $p_{c}$ is chosen as the representative of all the points, which can be calculated by Equation (1):

(1) $$p_{c}=\frac{1}{m}\overset{m}{\underset{i=1}{\sum }}p_{i}$$

where $p_{i}$ and m are the point and the number of points in a voxel, respectively.

In order to reduce the outliers and noisy points caused by light interference and silage splashing in the agricultural environment, we perform statistical filtering on the down-sampled point cloud using the statistical outlier removal algorithm [23]. Statistical filtering performs statistical analysis on adjacent points of each point and calculates its average distance to all adjacent points. Assuming that the result obtained is a Gaussian distribution whose shape is determined by the mean and standard deviation, the point whose average distance is outside the standard range can be defined as outlier and removed from the data [24]. For each point $p_{i}\left(x_{i},y_{i},z_{i}\right)\;$(j=1,2,…,n) in the point cloud, the algorithm calculate its mean distance ${\bar{d}}_{i}$ to the *k* nearest neighbors $p_{j}\left(x_{j},y_{j},z_{j}\right)\;$(j=1,2,…,k) through Equation (2). Assuming that the mean and standard deviation of the Gaussian distribution are μ and σ respectively. The mean μ and the standard deviation σ can be calculated by Equations (3) and (4):

(2) $${\bar{d}}_{i}=\frac{1}{k}\overset{k}{\underset{j=1}{\sum }}\sqrt{{\left(x_{i}-x_{j}\right)}^{2}+{\left(y_{i}-y_{j}\right)}^{2}+{\left(z_{i}-z_{j}\right)}^{2}}$$

(3) $$\mu =\frac{1}{n}\overset{n}{\underset{i=1}{\sum }}{\bar{d}}_{i}$$

(4) $$\sigma =\sqrt{\frac{1}{n}\overset{n}{\underset{i=1}{\sum }}{\left({\bar{d}}_{i}-\mu \right)}^{2}}$$

where *k* and n are the number of nearest neighbors and the number of the points in the entire point cloud, respectively.

The points whose mean distance is outside the range of [μ−ασ, μ+ασ] are defined as outliers and should be removed from the point cloud. The parameters α and *k* depend on the density of the points and actual requirements. The experimental test in this paper found that the filtering effect is best when α = 1.0 and *k* = 45.

#### 2.2.3. Point Cloud Pose Transformation

After preprocessing, the coordinates of the point cloud data are still based on the camera coordinate system. In order to transform the point cloud to the base coordinate system, this paper refers to the SVD algorithm in the field of visual odometry (VO) to solve the transformation relationship from the camera coordinate system to the base coordinate system. The transformation relationship contains two parts: rotation and translation. In a VO system, the basic task is to estimate the movement of the camera through image changes [25]. This type of motion estimation needs to extract and match the feature points between two consecutive images. Combined with the depth images, the 3D coordinates of those feature points can be determined. Through the SVD algorithm, the pose transformation relationship of the two sets of corresponding feature points can be effectively calculated. However, the transformation from the camera coordinate system to the world coordinate system in this paper is also similar to constructing a VO between two frames of images. In this case, the two sets of corresponding feature points are the 3D coordinates of the container’s selected reference points in the camera coordinate system and the base coordinate system, respectively. The coordinates of the reference points in the camera coordinate system are extracted from the point cloud, and the coordinates in the base coordinate system are directly measured. Supposing that *P’* is the feature points set in the camera coordinate system, and *P* is the corresponding feature points set in the base coordinate system:

(5) $$\{\begin{array}{c} P=\left(p_{1},p_{2},\ldots ,p_{n}\right)\\ P^{\prime }=\left(p_{1},p_{2},\ldots ,p_{n}\right) \end{array}$$

where *n* is the number of feature points. Two feature points sets are related by the rigid body transformation of the following form:

(6) $$\forall i,p_{i}=R{p^{\prime }}_{i}+t\;(i=1,\;2,\;\ldots ,\;n)$$

where $R\in {\mathbb{R}}^{3\times 3}$ is a rotation matrix, $t\in {\mathbb{R}}^{3\times 1}$ a translation vector, $p_{i}$ is a point in P, and ${p^{\prime }}_{i}$ is a point in *P’.*

We construct the following least squares problem to find the optimal *R* and *t* matrices that minimize the sum of squared errors *J* of two feature points sets:

(7) $$\underset{R,t}{\min}J=\frac{1}{2}\overset{n}{\underset{i=1}{\sum }}\|(p_{i}-\left(R{p^{\prime }}_{i}+t\right){\|}_{2}^{2}$$

The centroids of two sets of points are calculated by:

(8) $$\{\begin{array}{c} p=\frac{1}{n}\overset{n}{\underset{i=1}{\sum }}p_{i}\\ p^{\prime }=\frac{1}{n}\overset{n}{\underset{i=1}{\sum }}p{’}_{i} \end{array}$$

where *p* is the centroid of the points set *P*, and *p’* is the centroid of the points set *P’.* The de-centroid coordinates of each point can be calculated by:

(9) $$\{\begin{array}{c} q_{i}=p_{i}-p\;\\ q{’}_{i}=p{’}_{i}-p’ \end{array}$$

where $q_{i}$ is the de-centroid coordinate of the points set *P*, and $q{’}_{i}$ is the de-centroid coordinate of the points set *P’*. We define a matrix W∈SO(3) which is described in Equation (10):

(10) $$W=\overset{n}{\underset{i=1}{\sum }}q_{i}q{’}_{i}^{T}$$

According to the SVD algorithm, *W* can be decomposed into the following form:

(11) $$W=USV^{T}$$

where *S* is a 3 × 3 diagonal matrix composed of singular values, U and V are both 3 × 3 diagonal matrices. When *W* is full rank, the optimal solution of the rotation matrix *R* and the translation matrix *t* in Equation (6) can be expressed as:

(12) $$R=UV^{T}$$

(13) t=p−Rp’

These are also the rotation matrix Rcamerabase and the translation matrix tcamerabase from the camera coordinate system to the base coordinate system. Finally, we can transform the point cloud to the base coordinate system through Equation (14):

(14) Pb=RcamerabasePc+tcamerabase

where $P_{b}$ is the point cloud in the base coordinate system, $P_{c}$ is the point cloud in the camera coordinate system.

#### 2.2.4. Segmentation of the Upper Edge Points

In order to reduce the economic loss caused by the parking of the forage harvester, multiple trucks undertake the task of forage transportation. The height and size of different truck containers are different, and it is time-consuming and inefficient to manually measure the information of each container. However, the height value is necessary to segment the point cloud of the upper edge. Therefore, this section proposes a segmentation algorithm for the upper edge point cloud, which automatically measures the height of the container. The steps of the algorithm are as follows:

Step 1: Sort the point cloud $P_{b}$ in descending order of z-axis value to form a sequence of three-dimensional coordinate points.

Step 2: Set a sliding window with a length of 20 and a step length of 1. Start sliding from the head of the sequence, and calculate the variance of the z-axis values of the points of each window in turn. When the variance is less than the set threshold, the mean of the z-axis values of the 20 points in the window is the height h of the container. After the point cloud is sorted in descending order of z-axis values, the points of the upper edge are concentrated at the head of the sequence. The purpose of using the sliding window is to eliminate the interference of outliers and obtain more accurate height value.

Step 3: Set a reasonable z-axis value interval [h−a,h+a] and divide the entire point cloud $P_{b}$ according to this interval to obtain the upper edge point cloud $P_{cut}$ which only contains the upper edge points of the container.

Step 4: project $P_{cut}$ to the $X_{base}$ − $Y_{base}$ plane to obtain a 2D image of the upper edge, which completes the dimensionality reduction process.

In this section, the segmentation of the container’s upper edge is completed, and its 2D image is obtained. The above step 1 and step 2 are the processes of automatically obtaining the height of the container. Since the container’s height is constant, it is only necessary to measure the height once before the start of work or the truck is replaced. After completing the measurement process, the height will be stored in the program as a constant. When the algorithm is called again, only step 3 and step 4 will be executed. In step 3, the reason for setting the z-axis threshold interval is that the truck fluctuates up and down while moving [26]. A reasonable setting of a can ensure that the upper edge point cloud of the container is always within the threshold interval. In particular, the point cloud input in this algorithm is based on the base coordinate system, so the calculated height is also relative to the base coordinate system.

#### 2.2.5. Identification and Positioning of the Container

**Figure 4:**
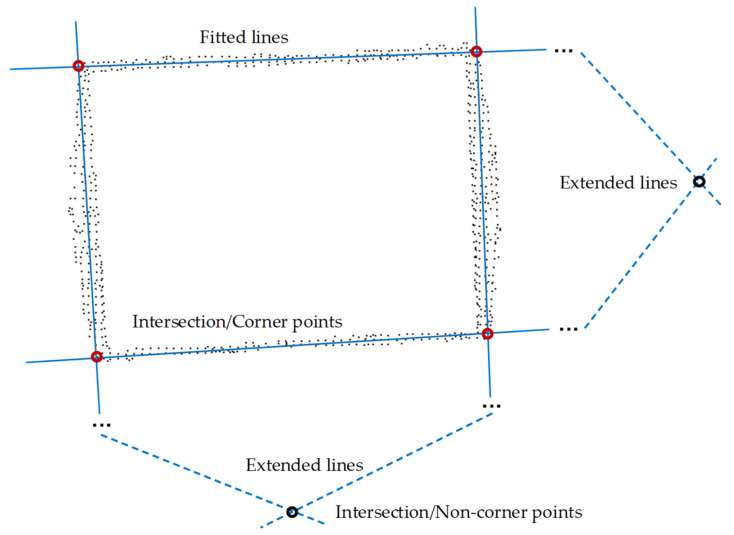
Schematic diagram of the fitting of edge lines and the positioning of corner points.

The purpose of identification is to determine the region of the container. The 2D image of the upper edge is composed of a large number of discrete points, as shown in Figure 4. Each edge has an apparently straight line feature, so four straight lines can be fitted from the 2D image. However, for each edge, the points belonging to this edge are inside points, while other points not belonging to this edge are outside points. Only by excluding these outside points can an accurate straight line be fitted. The least squares method is obviously not applicable, but the RANSAC algorithm has significant advantages for such problems [27,28]. The specific process of identifying the upper edge lines of the container is as follows:

Step 1: Input the 2D image and extract the four straight lines where the container’s upper edge is located using the RANSAC algorithm.

Step 2: Calculate and screen the six intersection points of the four straight lines. Since the straight lines are directly fitted from the data, the straight lines on the rectangle’s opposite sides are not completely parallel. The screening is based on the distance between the intersection of the straight lines and the origin. The intersections of the straight lines fitted by the two sets of opposite sides are the farthest from the origin, and they are eliminated.

Step 3: Connect the remaining four corner points in order of coordinates according to the characteristics of the rectangle so that the container region is identified.

The above process has solved the sideline equations and the x-axis and y-axis values of the upper edge’s corner points in the base coordinate system. This realizes the positioning of the container in the $X_{base}$ − $Y_{base}$ plane. Since the z-axis value of the container height has been obtained in Section 2.2.4. So far, the spatial region of the upper edge can be determined, and the spatial positioning of the container in the base coordinate system has been completed.

#### 2.2.6. Visualization of the Results

The visual feedback of the identification results is helpful for operators to monitor the unloading status of forage in real-time. This paper uses an inverse coordinate transformation to transform the calculated corner points and edge lines into the camera coordinate system, maps them to the pixel coordinate system through the camera internal parameter matrix, and then marks the region of the container on the RGB image or the depth image. Finally, the results will be displayed on the vehicle monitor.

By executing Equation (15), the coordinates of the corner points in the base coordinate system can be inversely transformed to the camera coordinate system:

(15) Pc=RcamerabaseTPb−RcamerabaseTtcamerabase

Then the corner points are transformed into the pixel coordinate system through the camera internal parameter matrix. According to the pinhole camera model, the transformation equation is as follows:

(16) $$P_{uv}=\left[\begin{array}{c} u\\ v\\ 1 \end{array}\right]=\frac{1}{z}\left[\begin{array}{ccc} f_{x} & 0 & c_{x}\\ 0 & f_{y} & c_{y}\\ 0 & 0 & 1 \end{array}\right]\left[\begin{array}{c} x\\ y\\ z \end{array}\right]=\frac{1}{z}KP_{c}$$

where $P_{uv}$ is the homogeneous coordinate of the corner point in the pixel coordinate system, $f_{x}$ and $f_{y}$ are focal length parameters, $c_{x}$ and $c_{y}$ are principal point offset parameters, *K* is the camera internal parameter matrix which is only determined by the camera’s internal parameters.

## 3. Field Experiment and Results Analysis

### 3.1. Field Experiment Site and Equipment

**Figure 5:**
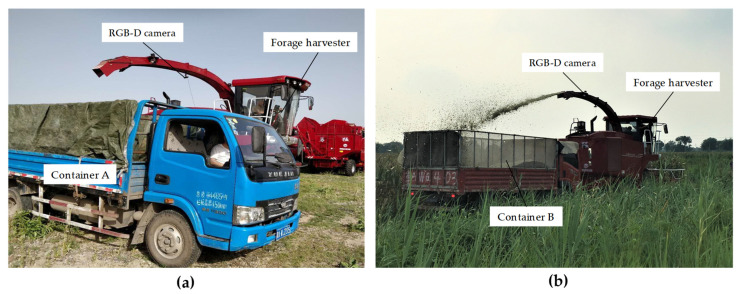
The identification and positioning experiments with different trucks. (**a**) Container A is heightened by welding steel and covered by a tarpaulin. (**b**) Container B is modified from the high-strength mesh.

The field experiment was conducted on a commercial farm in northern Tianjin, China during August 2019. The crop grown on the farm was corn silage, with a plant height of about 1.6 m. The RGB-D camera was fixed on the discharge elbow of the forage harvester. The composition of the entire system and the coordinate systems’ settings were exactly the same as those described in Section 2.2.1. To improve transportation efficiency, several trucks are generally used to transport forage during harvesting, and the appearance of these truck containers may vary dramatically from machine to machine. Therefore, two trucks with different container types were prepared. Container A is heightened by welding steel, and the tarpaulin is covered inside to reduce the spillage of forage and facilitate unloading, as shown in Figure 5a. The size of container A is 3.50×2.00×2.25 m. Container B is modified from high-strength mesh, with small surface pores, lightweight and low cost, as shown in Figure 5b. The size of container B is 3.75×2.25×2.10 m. These are two common types of forage transport containers in agriculture.

The RGB-D camera used in the experiment is FM810-5M (Percipio Co., Ltd., Shanghai, China). The measurement distance is 0.5–6 m, and the measurement error is 0.25% of the measurement distance. The RGB image resolution is 640 × 480 pixels, the depth image resolution is 560 × 460 pixels, and the frame rate is 15fps. The forage harvester model is Yongmeng 9QS-300 (Yongmeng Machinery Co., Ltd., Tianjin, China), the cutting width is 3000 mm, and the operating speed is 6km/h. All algorithms in this paper were developed by C and C++ under CLion 2020.3 environment (Jetbrains sro, Prague, Czech Republic) in an experimental computer equipped with Intel(R) i7-6500 CPU @3.40 GHz, 8 GB of RAM, running on Linux Ubuntu 16.04 LTS. The libraries that the algorithm mainly relied on were OpenCV 3.4.10, PCL 1.7 and Eigen 3.3.7.

### 3.2. Evaluation Metrics of the CAIP Method

The performance of the approach is evaluated from two aspects: The Intersection-over-Union (*IoU*) of the identification region of the container and its positioning accuracy. The *IoU* is the most popular evaluation metric for object detection and semantic segmentation [29], which is formulated as:

(17) $$IoU=\frac{\left|A\;\cap^{\;}B\right|}{\left|A\;\cup^{\;}B\right|}$$

where *A* denotes the total number of pixels in an image that are predicted as the container area by the algorithm; *B* is the total number of pixels in the real container area calibrated manually.

The identification result of the truck container is a spatial plane area, and it is very difficult to directly measure the positioning accuracy of this area. However, the final purpose of the positioning is to determine the ideal unloading point coordinates of the forage in the container, and the ideal unloading point is generally selected at the center point of the container area [15]. Therefore, this paper selects the absolute error between the predicted value and the theoretical value of the center point coordinate to evaluate the positioning accuracy [30]. Based on this criteria, the absolute positioning error e of the center point is described as follows:

(18) $$e=\|C_{p}-C_{t}\|=\sqrt{{\left(x_{p}-x_{t}\right)}^{2}+{\left(y_{p}-y_{t}\right)}^{2}+{\left(z_{p}-z_{t}\right)}^{2}}$$

where $C_{p}=\left(x_{p},y_{p},z_{p}\right)$ and $C_{t}=\left(x_{t},y_{t},z_{t}\right)$ are the coordinates of the predicted center point and the theoretical center point of the container, respectively.

### 3.3. Results Analysis and Discussion

In the collaborative operation of the forage harvester and the truck, due to speed differences or some special circumstances, such as turning, the relative position and distance between the two vehicles are not entirely constant. To comprehensively evaluate the performance of the approach proposed in this paper, the forage harvester traveled in a straight line at a rated working speed of 6 km/h, the following speed and direction of the truck were adjusted appropriately to enable the camera to capture data of containers in different positions. During the experiment, containers are always within the measuring range of the RGB-D camera.

**Figure 6:**
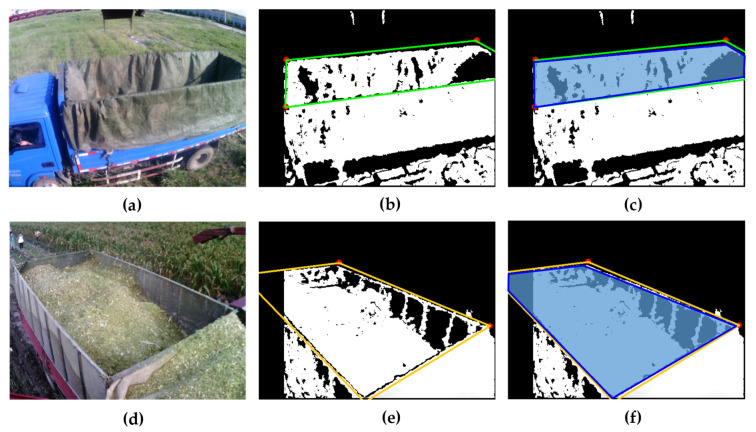
Identification results of two different containers. (**a**) RGB image of the container A; (**b**) Visualization of the identification results of the container A on the depth image; (**c**) The *IoU* calculation area of the container A, where the blue-purple area with a blue border represents the real container area. (**d**) RGB image of the container B; (**e**) Visualization of the identification results of the container B on the depth image; (**f**) The *IoU* calculation area of the container B, where the blue-purple area with a blue border represents the real container area.

Figure 6 shows the identification results of the containers of two different trucks. Table 1 selects the *IoU* data corresponding to 15 consecutive images to evaluate container area identification accuracy.

**Figure 7:**
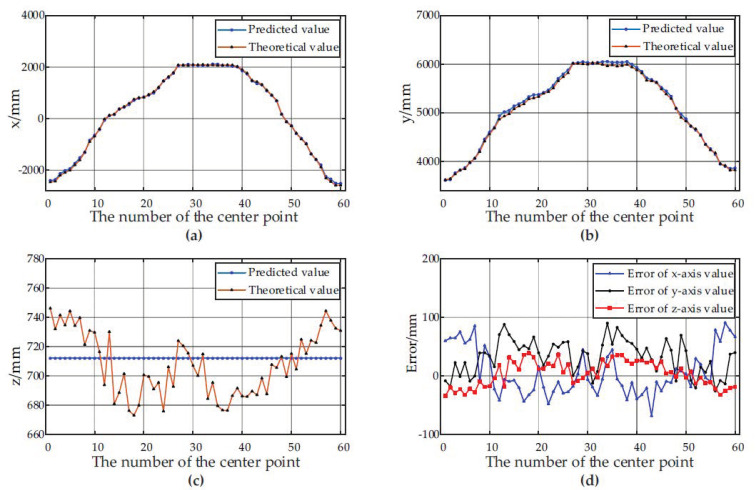
Positioning results of the center point of the container A at 60 different positions. (**a**) The predicted and theoretical values of x axis; (**b**) The predicted and theoretical values of y axis; (**c**) The predicted and theoretical values of z axis. (**d**) The error between the predicted values and the theoretical values at different positions.

**Figure 8:**
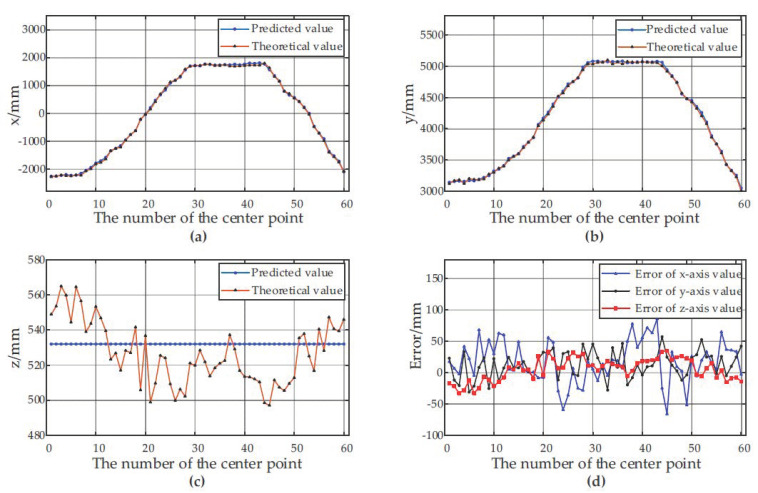
Positioning results of the center point of the container B at 60 different positions. (**a**) The predicted and theoretical values of x axis; (**b**) The predicted and theoretical values of y axis; (**c**) The predicted and theoretical values of z axis. (**d**) The error between the predicted values and the theoretical values at different positions.

Figure 7 and Figure 8 show the predicted values, theoretical values and errors of the center point coordinates of the container A and the container B at 60 different positions relative to the forage harvester.

**Figure 9:**
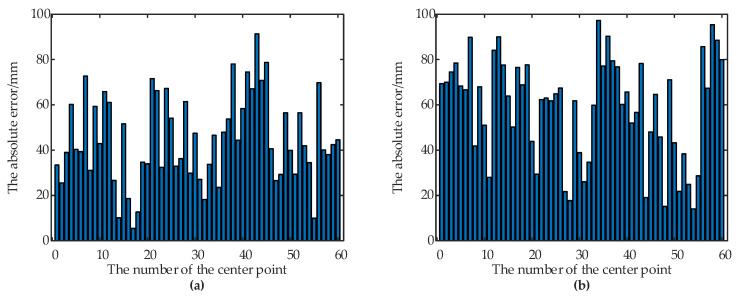
The positioning accuracy of the center point of the two containers. (**a**) The absolute errors of the center point of the container A. (**b**) The absolute errors of the center point of the container B.

According to the experimental results, the depth-vision-based CAIP algorithm can effectively identify and position two kinds of containers with different appearances. Figure 6 and Table 1 show that the average *IoU* for the container A is 90.69%, and the average *IoU* for the container B is 89.56%. Figure 7 and Figure 8 show the predicted and theoretical x-axis values, y-axis values, z-axis values and errors of the center points of the container A and the container B at 60 different positions. Furthermore, the center point’s absolute positioning errors are shown in Figure 9, which are calculated by Equation (13). To quantify and analyze the results of the positioning experiments, Table 2 provides quantitative statistics on the absolute errors and their component errors on the x-axis, y-axis and z-axis of the center points. For the container A, the maximum absolute error of positioning is 97.22 mm, the mean is 58.88 mm, and root-mean-square error (RMSE) is 23.85 mm. For the container B, the maximum absolute error is 91.36 mm, the mean is 44.62 mm, and RMSE is 26.56 mm. The error components on the three coordinate axes appear to be smaller, the error of x-axis values is less than 90 mm, the error of y-axis values is less than 90 mm, and the error of z-axis values is less than 40 mm. The experimental results are stable for 6 tests in the field. Reference 16 provided an another container positioning method based on the laser sensor and GNSS [16], the positioning errors are: maximum error of (161 mm, 116 mm, 65 mm), mean error of (68 mm, 60 mm, 26 mm), and RMSE of (83 mm, 67 mm, 32 mm) in x, y, and z axes, respectively. Compared with Table 2, the method proposed in this paper has better positioning accuracy.

Figure 7c and Figure 8c show that the predicted values of the z axis of the center point are always constant, while the theoretical values are constantly fluctuating. The predicted value of the z axis of the center point is the height value of the container. The height is calculated before the work and remains unchanged throughout the harvesting process. However, the theoretical value fluctuates by ±50 mm, the reason is that the unevenness of the farmland causes the truck to bump up and down, thus changing the height of the upper edge of the container. Therefore, it is crucial to set a reasonable variable a to extract the upper edge the upper edge point cloud in Section 2.2.4. On the one hand, when the vehicle turbulence is severe but the value of the variable a is too small, it may cause the upper edge points of some frames to be outside the interval [h−a, h+a], resulting in failure to extract the upper edge of the container. On the other hand, if the variable a is set too large, it may introduce other interference, such as the point cloud of the truck cab, affecting the accuracy of the algorithm.

Although the appearance of the containers has significantly changed due to artificial modification, as shown in Figure 6a,d, there are always two common features that have not changed: (1) The edge of the container has obvious straight-line characteristics. (2) The container’s upper edge is on a spatial plane and is located at the local highest position of the truck. Therefore, the method in this paper is not sensitive to the appearance of container and has good versatility. In addition, because the two vehicles are independently controlled during operation, the container information obtained by the camera may be incomplete. As long as the point cloud data contains the local information of the four upper edge lines, the container can also be identified and positioned. As shown in Figure 6b,e, both containers A and B lack the information of one corner, under such circumstances the containers are still effectively identified and positioned.

## 4. Conclusions and Future Work

In this paper, a model with three coordinate systems is established to describe a collaborative harvesting system, and a method is proposed to identify and position the truck container based on depth vision. The method processes include data preprocessing, point cloud pose transformation based on the SVD algorithm, segmentation and projection of the upper edge, edge lines extraction and corner points positioning based on the RANSAC algorithm, and fusion and visualization of results on the depth image. The effectiveness of the proposed method has been verified by field experiments. The results show that the depth-vision-based CAIP method can effectively identify and position two kinds of containers with different appearances. The identification accuracy of the container region is about 90%, and the absolute error of center point positioning is less than 100 mm. In addition, the method is not sensitive to the appearance of the truck container and noisy points in the agricultural environment, and performance is relatively stable, which can meet the requirements of dynamic identification and positioning of containers in forage harvesting.

The theories on the identification and positioning method of the transport vehicle containers have laid the foundation for the automation of the unloading system of the forage harvester. In the future, we will focus on the autonomous driving and path planning of the unloading mechanism of the forage harvester.

## Figures and Tables

**Table 1:** The *IoU* data of 15 consecutive images of two containers.

Container Type	Image No.	Union/Pixels	Intersection/Pixels	*IoU*	Average of *IoU*
Container A	1	69474	63180	0.9094	0.9069
	2	70520	64258	0.9112	
	3	72525	66331	0.9146	
	4	71343	65643	0.9201	
	5	74794	67015	0.8960	
	6	71794	63854	0.8894	
	7	72794	65595	0.9011	
	8	75297	67105	0.8912	
	9	71352	66329	0.9296	
	10	69400	62994	0.9077	
	11	72093	64588	0.8959	
	12	72539	66337	0.9145	
	13	73709	67982	0.9223	
	14	76293	67237	0.8813	
	15	71228	65444	0.9188	
Container B	1	147787	135003	0.9135	0.8956
	2	156686	138087	0.8813	
	3	152595	133139	0.8725	
	4	156609	141512	0.9036	
	5	161556	147064	0.9103	
	6	157791	139408	0.8835	
	7	156961	141438	0.9011	
	8	166250	149475	0.8991	
	9	146632	133655	0.9115	
	10	153721	141931	0.9233	
	11	149072	132570	0.8893	
	12	160023	134659	0.8415	
	13	148056	134050	0.9054	
	14	159701	143843	0.9007	
	15	151321	135856	0.8978	

**Table 2:** Quantitative statistics of positioning errors.

Container Type	Error	Maximum Value/mm	Minimum Value/mm	Average Value/mm	RMSE/mm
Container A	x	90.76	−68.66	5.77	39.53
	y	90.10	−25.88	32.48	29.16
	z	38.99	−34.24	4.10	21.23
	Absolute error	97.22	14.04	58.88	23.85
Container B	x	85.70	−66.06	13.04	33.62
	y	57.09	−31.12	13.01	21.14
	z	34.91	−32.96	5.41	17.51
	Absolute error	91.36	5.43	44.62	26.56

## Data Availability

Not applicable.
